# Olefin Metathesis Reaction in Water and in Air Improved by Supramolecular Additives

**DOI:** 10.3390/molecules201019130

**Published:** 2015-10-21

**Authors:** Jasmine Tomasek, Miriam Seßler, Harald Gröger, Jürgen Schatz

**Affiliations:** 1 Organic Chemistry 1, Department of Chemistry and Pharmacy, Friedrich-Alexander-Universität Erlangen-Nürnberg (FAU), Henkestraße 42, D-91054 Erlangen, Germany; E-Mails: jasmine.tomasek@fau.de (J.T.); m.b.sessler@web.de (M.S.); 2 Faculty of Chemistry, Bielefeld University, Universitätsstraße 25, 33615 Bielefeld, Germany; E-Mail: harald.groeger@uni-bielefeld.de

**Keywords:** aqueous metathesis, supramolecular chemistry, C-C coupling

## Abstract

A range of water-immiscible commercially available *Grubbs*-type precatalysts can be used in ring-closing olefin metathesis reaction in high yields. The synthetic transformation is possible in pure water under ambient conditions. Sulfocalixarenes can help to boost the reactivity of the metathesis reaction by catalyst activation, improved mass transfer, and solubility of reactants in the aqueous reaction media. Additionally, the use of supramolecular additives allows lower catalyst loadings, but still high activity in pure water under aerobic conditions.

## 1. Introduction

In organic chemistry, C-C coupling reactions open a wide range of applications for effective synthesis, which otherwise would be difficult or even hardly feasible. The olefin metathesis reaction displays one of these atom efficient catalysis reactions under mild conditions [1]. Along a plethora of organometallic reactions, which are viable in aqueous media and do not need inert conditions or even a glove box [2,3], several research groups have intensively studied aqueous metathesis reaction. Two main strategies can be distinguished: either the design of water-soluble catalysts to obtain homogeneous conditions, or using water-immiscible commercially available catalysts to benefit from the advantages of heterogeneous conditions [4,5,6,7,8,9,10]. In the latter case, metathesis can be performed in pure water, in homogeneous aqueous solvent-mixture [11,12,13] by using special methods such as microwave [14] or ultrasonic irradiation [15], and with the aid of additives or catalyst ligands with and without micelle character [10].

Recently, we investigated the influence of various non-amphiphilic supramolecular additives in ring closing metathesis (RCM) and cross metathesis (CM) reactions performed in pure water and catalysed by commercial available *Grubbs* type catalysts [16]. In the RCM of *N*-tosyldiallylamine, *p*-sulfocalixarenes (**1**) in combination with the second-generation *Grubbs* catalyst emerge as one of the most efficient amongst all tested supramolecular additives, which enhance metathesis activity from 75% without to 99% conversion with an additive. Another positive effect of these water-soluble additives is a type of “solubilisation” of the catalytic species. By addition of sulfocalixarenes **1**, a heterogeneous mixture is converted to a pseudo-homogeneous mixture, visible by the naked eye. This effect might be a reason for enhanced metathesis activity. As we know from our own measurements, water-soluble calixarene derivatives can form supramolecular host-guest complexes with non-charged phenyl-derivatives of a suitable size [17]. Monflier *et al*. showed that in a comparable catalytic system, the aqueous hydroformylation reaction, cyclodextrins as supramolecular additives promote the formation of catalytic active species [18,19]. To get a more predicating explanation besides the visible effect, we focused our research on the interaction of the catalyst compounds, the substrate and the supramolecular additive.

## 2. Results and Discussion

### 2.1. Catalyst Screening

**Figure 1:**
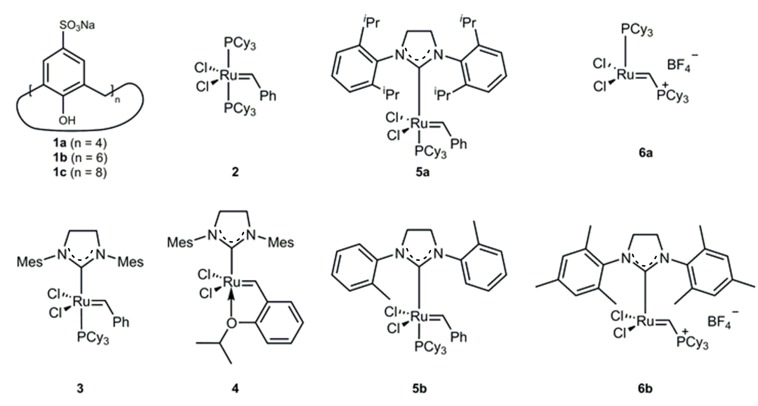
Supramolecular additive *p*-sulfocalixarenes **1** and *Grubbs* type (pre)catalysts used in aqueous ring closing metathesis (RCM) reactions.

We studied the catalytic activity in aqueous media of different commercially available *Grubbs* type catalysts (Figure 1) in standard RCM reactions of *N*-tosyldiallylamine and diallylmalonate as substrates. All reactions were performed under standardized reaction conditions, *i.e.*, room temperature, air-atmosphere and pure water as solvent to get a comparable insight into the reactivity of various catalyst precursors. The work was not targeted towards optimization of reaction conditions. After a defined reaction time, the conversion values were determined by ^1^H-NMR spectroscopy (Table 1). Besides well-known *Grubbs* precatalysts **2**, **3** and **4**, also precatalyst **5a** and NHC-bearing, cationic catalyst **6b** showed efficient metathesis reactions in pure water with conversion ranging from 75% up to quantitative yields. Metathesis activity increases with the increase of the percent buried volume of NHC ligands *o*-SIMe < SIMes < *o*-SIPr [20]. Comparison of the phosphine bearing precatalysts **2** and **6a** show completely opposing results. Catalysis using 5-coordinated precatalyst **2** almost ends up in complete conversion of substrate **7** to the cyclic product, while 4-coordinated **6a** is inactive in the metathesis reaction performed in water of both **7** and **9**. To verify the reason for the inactivity, reactions were performed under Ar-atmosphere (Entry 13) and exclusion of water (Entry 14) with reaction parameters comparable to literature [21]. However, no improvements in catalytic activity of **6a** were achieved, probably due to sensitivity of the catalyst.

This catalyst screening is another example for efficient metathesis reactions performed in water under aerobic conditions using commercially available *Grubbs* type catalysts [3,10].

**Table 1:** Results of aqueous RCM reactions of substrate **7** and **9** induced by various *Grubbs* type (pre)catalysts **2**–**6**. 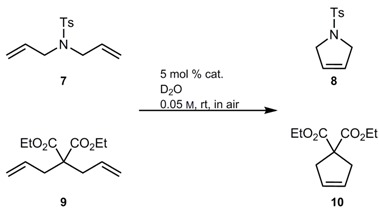

Entry	Substrate	*Cat.*	t	Conversion [%]
1	**7**	**2**	4 h	95
2	**7**	**3**	4 h	75
3	**7**	**4**	4 h	89
4	**7**	**5a**	4 h	83
5	**7**	**5b**	4 h	5
6	**7**	**6a**	5 h	3
7	**7**	**6b**	4 h	97
8	**9**	**4**	1 h	>99
9	**9**	**5a**	5 min	>99 ^a^
10	**9**	**5b**	4 h	36
11	**9**	**6a**	21 h	3
12	**9**	**6b**	4 h	84
13	**9**	**6a**	2 h	0 ^b^
14	**9**	**6a**	2 h	7 ^c^

^a^ metathesis reaction performed once. ^b^ 0.23 M, 1 mol % **6a**, Ar-atmosphere. ^c^ 0.23 M, 1 mol % **6a**, CD_2_Cl_2_.

### 2.2. Solubilisation

**Figure 2:**
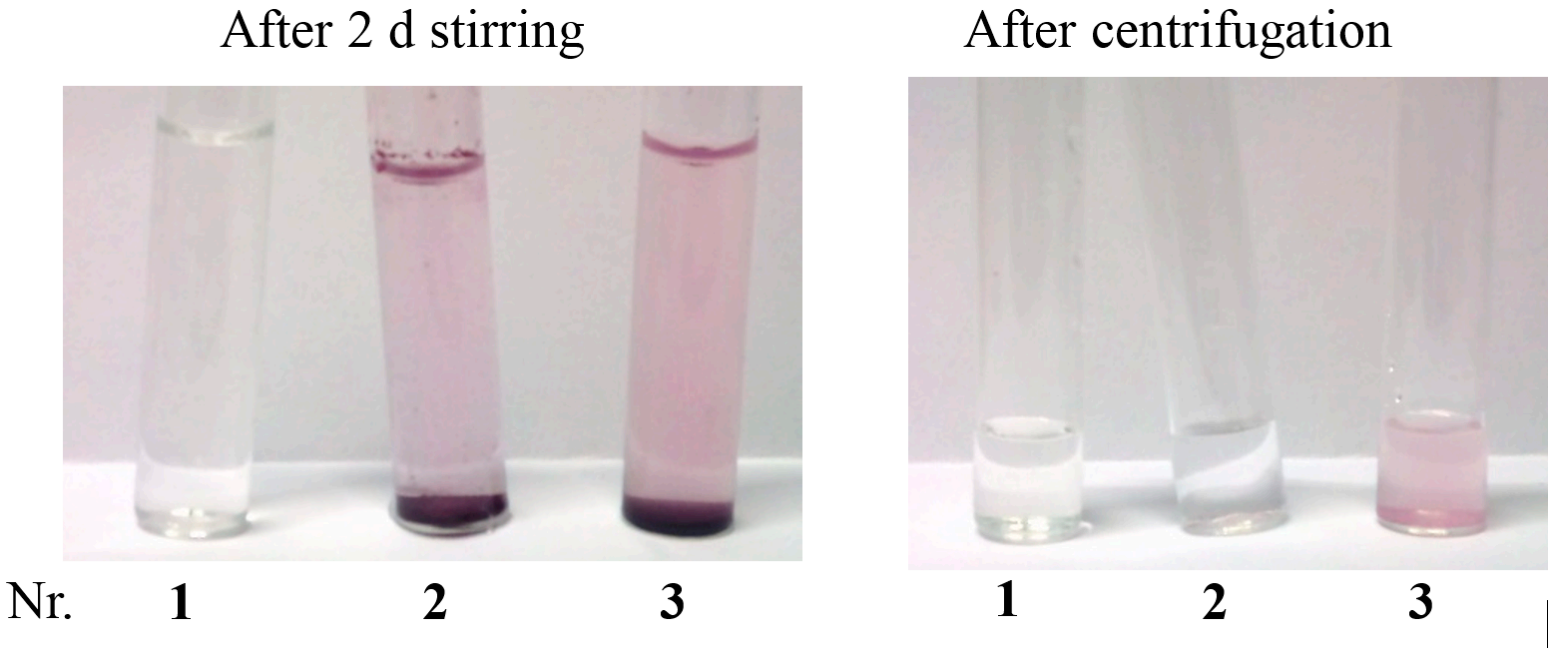
(Micro)Solubilization effect in pure water: sulfocalix[4]arene **1a** (sample 1); *Grubbs* I **2** (sample 2); sulfocalix[4]arene **1a**, *Grubbs* I **2** (sample 3).

Both precatalysts, *Grubbs* first (**2**) and second (**3**) generation, showed the aforementioned solubilisation effect by addition of sulfocalixarene **1** in water (Figure 2) [16]. We supposed that catalytic species derived from **2** are present in solution and can therefore be analyzed by NMR or UV/Vis spectroscopy. However, in both cases, ^31^P-NMR or UV/Vis spectroscopy could not detect any catalytic species in water. This indicates that the concentration of the active catalyst species in water is smaller than approx. 10^−5^ M. To get a better insight in the additive effect, the same experiments were performed in MeOD-*d*_4_. In ^31^P-NMR spectra, signals of protonated *P*-species HPCy_3_^+^ as well as the oxidized species O=PCy_3_ are visible (*cf.* Supplementary Materials). A comparison of the sample with and without additive showed that sulfocalixarene enhances the phase transfer of these P-species in solution. These measurements indicate that added sulfocalixarenes does not entirely dissolve the (pre)catalyst leading to only partially homogenous conditions.

### 2.3. Mass Transfer Effect

**Figure 3:**
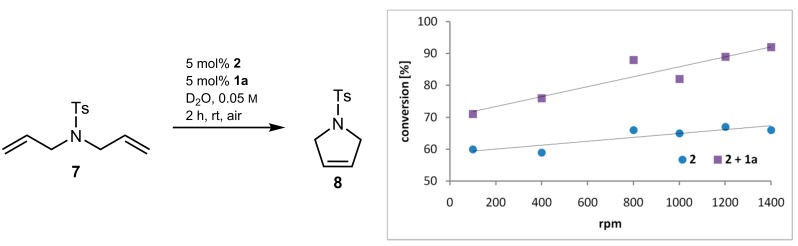
Dependency of the stirring speed on the catalytic activity in RCM of **7**; without additive (blue dots), with additive **1a** (purple squares).

In further experiments, we tested for a mass transfer effect, affected by sulfocalix[4]arene in aqueous media. In a standard RCM reaction of *N*-tosyldiallylamine (**7**) catalysed by *Grubbs* precatalyst of the first generation (**2**), experiments were performed with varying stirring speed, and in the presence or in the absence of sulfocalixarene **1a** (Figure 3). Apart from this, all reactions were performed under aforementioned standardized reaction conditions for 2 h. In experiments without additive, the conversion values hardly differ with varying stirring speed from 60% at 100 rpm to 66% at 1400 rpm. There is nearly no dependency of the stirring speed on the catalytic activity. In contrast, addition of sulfocalixarene to the reaction mixture indicates distinct dependency between stirring rate and conversion. The higher the stirring speed is the higher is the conversion value, with an increase of 21% from 100 rpm to 1400 rpm. This increase indicates that sulfocalixarene promote mass transfer in the reaction media.

### 2.4. Homogeneous vs. Heterogeneous Conditions

**Figure 4:**
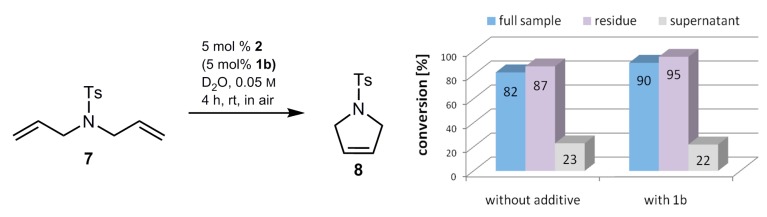
Conversion values of homogeneous (grey bars) and heterogeneous (purple bars) conditions in RCM of substrate **7** in D_2_O.

To check the catalytic activity of a pure homogeneous phase, a mixture of the precatalyst, sulfocalixarene, when applicable, and D_2_O (400 µL) was stirred in an Eppendorf tube for 14 h and subsequently centrifuged. The aqueous supernatant (350 µL) was separated from the solid residue (50 µL) and both samples were used in a catalytic reaction under our standard reaction conditions. After a reaction time of 4 h, the conversion values were determined by ^1^H-NMR spectroscopy (Figure 4).

In all experiments, only a small part of the precatalyst gets lost during the transfer of the supernatant into another reaction vessel. In both cases, with or without the additive **1b**, catalytic conversion of the substrate **7** took place in the residue under heterogenic conditions as well as in the supernatant of the aqueous reaction mixture under homogeneous reaction conditions. As expected, the main catalytic reaction with four times higher conversion values occurs under heterogenic, surface-dependent conditions. In catalytic reactions using the supernatant of the reaction mixture, at least 22%–23% of the substrate is converted. This is a surprisingly high activity taking into account that the concentration of catalytic species in homogeneous solution is lower than 10^−5^ M. However, the values of samples with and without the additive do not differ significantly. The full catalytic system consists of a homogeneous part, which accounts for around one quarter of the conversion, and, potentially to a significant extent, catalysis at solid/solution interfaces.

### 2.5. Influence of Additive Concentration

**Figure 5:**
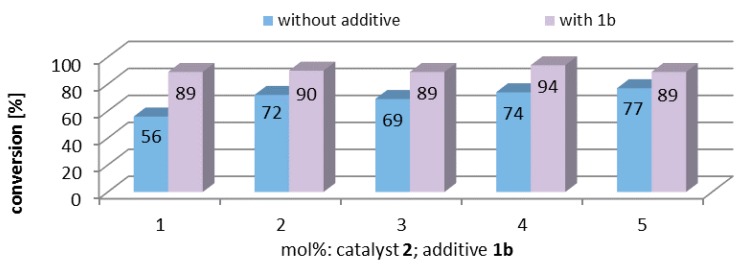
RCM conversion values of substrate **7**, by varying amount of catalyst **2** and concentration of additive **1b** concentration under standard reaction conditions: 0.05 M, 4 h, rt, air.

The aforementioned experiments show, that the amount of catalytic species in the aqueous phase is very low and sulfocalixarenes increase the catalytic activity. To check the relation between both phenomena, catalytic experiments with varying catalyst and/or substrate concentration were performed (Figure 5). The experimental series without additive (blue bars) show a decrease of the conversion values by lowering the amount of the precatalyst **2**, from 77% to 56% applying 1 mol % precatalyst. In contrast, in experimental series with precatalyst **2** and additive **1b** (purple bars) the catalytic activity stays constant with a conversion value ~92%. This means that commercially available sulfocalixarenes can offset the mentioned loss of catalytic activity and therefore a lower catalyst loading is possible.

### 2.6. Binding Studies

**Figure 6:**
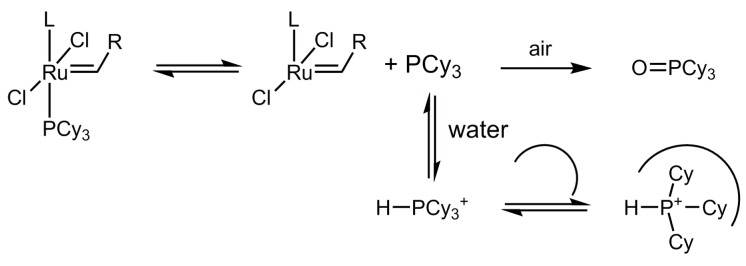
Supramolecular additives **1** (semicircle) as phosphine scavenger.

To get a better insight into the interaction of the (pre)catalyst, substrate, and additive, binding studies using ^1^H-NMR titration experiments were performed. Metathesis catalysts were mainly added as precatalysts, which form the catalytic active 14e^−^-species by a ligand dissociation step [1]. The NMR study suggests that the phosphine ligand released from *Grubbs* I **2** and II **3** precatalysts is protonated to some extent in aqueous media to HPCy_3_^+^ and/or gets oxidized to O=PCy_3_. To prove our assumption that sulfocalixarene **1** can act as a phosphine scavenger during the reaction and increase the catalytic activity, titration experiments were performed by varying concentration of sulfocalixarene **1** and constant HPCy_3_^+^ concentration in D_2_O and 7 µL of MeOD-*d*_4_ as internal standard (Figure 6 and Table 2) [22].

The calculated association constants *K*_ass_ are in a range of 7 × 10^4^ M^−1^ for calix[4]arene **1a** and 1 × 10^4^ M^−1^ for calix[6]arene **1b**, increasing with decreasing cavity size. Additionally to a cavity effect of sulfocalixarenes **1**, strong ionic interactions between cationic phosphonium-species and polyanionic sulfocalixarene lead to these high values, comparable to literature values of comparable systems (Table 2) [23,24,25,26,27].

**Table 2:** Log *K*_as*s*_ or *K*_ass_ and Δδ values (calculated by HypNMR) of receptors **1** with the guest **7**, **11** and **12** (^1^H-NMR, D_2_O, room temperature).

Guest	Host	log *K*_ass_	Δδ (ppm)
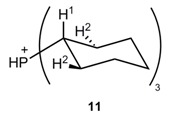	**1a**	4.9 ± 0.1	−0.42 (H^1^)
			−0.50 (H^2^)
	**1b**	4.0 ± 0.1	−0.54 (H^1^)
			−0.58 (H^2^)
	**1c**	log *K*_1_ = 4.4 ± 0.1	−0.61 (H^1^)
		log *K*_2_ = 7.3 ± 0.1	−0.59 (H^2^)
Guest	Host	*K*_ass_ (M^−1^)	Δδ (ppm)
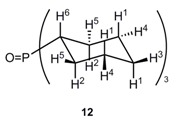	**1b**	29 ± 1	−0.54 (H^1^)
			−0.39 (H^2^)
			−0.94 (H^3^)
			−0.67 (H^4^)
			−0.40 (H^5^)
			−0.29 (H^6^)
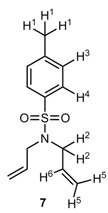	**1a**	22	−0.66 (H^1^)
			+0.25 (H^2^)
			−0.11 (H^3^)
			+0.19 (H^4^)
			+0.26 (H^5^)
			+0.30 (H^6^)
	**1b**	31	−0.40 (H^1^)
			−0.11 (H^2^)
			−0.22 (H^3^)
			−0.02 (H^4^)
			−0.17 (H^5^)
			−0.11 (H^6^)

**Figure 7:**
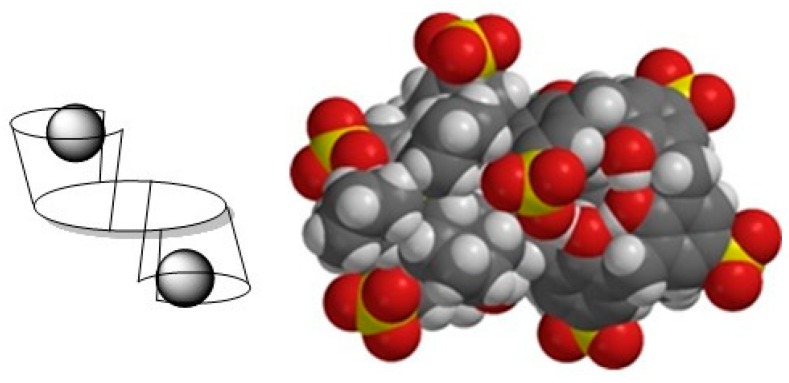
Schematic representation of inverted double partial cone conformation (**left**), PM3 optimization of 1:2 complex of host **1c** with guest **11** (**right**).

While sulfocalixarenes **1a** and **1b** with HPCy_3_^+^ fit to a 1:1 complex binding motif, complexes of **1c** and HPCy_3_^+^ result in a 1:2 complex. In the case of **1a** and **1b** the Ar-C*H*_2_-Ar ^1^H-NMR signal of the hosts, changes from a broad singlet to a sharp doublet. Obviously, the complexation process leads to a higher rigidity of the calixarene-scaffold; the stabilized cone conformation is clearly indicated by the reappearance of the typical AB-type NMR splitting pattern. In the case of calix[8]arene **1c**, *K*_2_ > *K*_1_ and *K*_1_ is in the same range as *K*_ass_ of calix[4]arene **1a**. Comparison with literature leads to the assumption of an inverted double partial cone conformation of the sulfocalixarene **1c** [28]. To support this, molecular modeling was performed (Figure 7) indicating an inclusion of two HPCy_3_^+^ cations in two half rooms formed by the calix[8]arene. The first complexation process helps to preorientate the receptor molecule for the second binding process resulting in high association constants for both processes. Attempts to prove that by an X-ray analysis failed up to now.

Not only ionic guests, but also non-charged, phenyl-bearing components can occupy the sulfocalixarene cavity. Further NMR titration experiments were conducted to identify any supramolecular interaction between sulfocalixarene **1** and the substrate *N*-tosyldiallylamine (**7**) in D_2_O and a small amount of DMSO as internal standard. In another experiment oxidized phosphine species Cy_3_P=O **12** in D_2_O and MeOD as internal standard were used. In both titration experiments, the guest concentrations were kept in a relatively low range from 10^−4^ to 10^−3^ M^−1^, because of solubility limitations. The results of serial dilution experiments of both the substrate **7** and the calixarenes **1a** and **1b**, confirm that self-aggregation of the compounds can be excluded.

In the case of non-charged guests **12** and **7**, C-H/π interactions are leading to a 1:1 binding motif and *K*_ass_ values are approximately 30 M^−1^, which fit well with literature values of comparable systems, sulfocalix[4]arene as host and phenyl-bearing guests (Table 2) [17]. The interaction between protonated phosphine ligand **11** and host **1** is 10^3^-times stronger, than interactions with irreversible oxidized phosphine species **12**, so HPCy_3_^+^ will win the competition in aqueous solution. In both titration experiments, all H-atoms showed an up-field shift (Δδ < 0), indicating an inclusion of the cyclohexyl-moiety into the hydrophobic pocket of calixarene **1**.

In the complexation experiments of sulfocalixarenes **1** and the substrate **7**, *K*_ass_ values are again low, but the position of the substrate in the calixarene pocket differs. Sulfocalix[6]arene with the bigger cavity incorporates the whole molecule, not in a linear way, but more probably angled, to fit the aromatic ring and the allyl groups in the cavity. In contrast, the smaller cavity of sulfocalix[4]arene incorporates only the aromatic H-atoms H^1^ and H^3^ of **7**, while protons of the allyl groups are placed outside the hydrophobic pocket.

**Figure 8:**
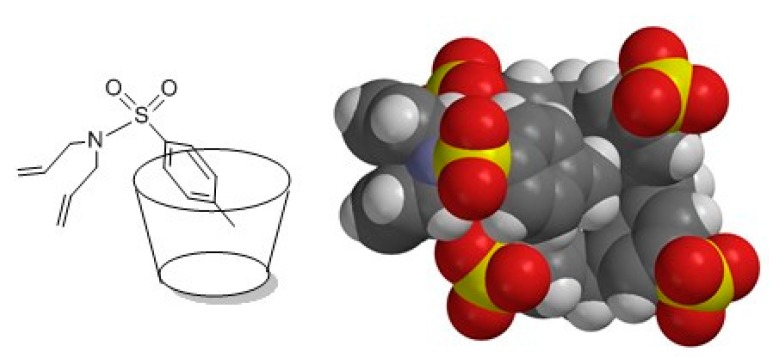
Schematic representation of complex conformation (**left**), MMFF optimization of 1:1 complex of host **1a** with guest **7** (**right**).

These supposed complex conformations are in agreement with calculated MMFF optimizations (Figure 8). The obtained complex induced ^1^H shift of the methyl group pointing inside the cavity for the optimized conformation of 0.62 ppm (analogously to a published procedure [29]) also does not differ significantly from the experimental value of 0.66 ppm.

## 3. Experimental Section

All chemicals, precatalysts **2**–**6**, sulfocalixarene **1** and substrate Diethyl diallylmalonate **9** were purchased from Acros^®^, Aldrich^®^, Merck^®^ or VWR^®^ and used without further purification, unless otherwise specified. Substrate *N*-tosyldiallylamine **7** was synthesized according to a literature procedure [30]. All NMR spectra were recorded at room temperature (298 K) on a BRUKER Avance 400 spectrometer. Chemical shifts (δ) are expressed in ppm and refer to the not-deuterated amount of used solvents [δ_H_ (CDCl_3_) = 7.26, δ_H_ (D_2_O) = 4.79, δ_H_ (MeOD-*d*_4_) = 3.31, δ_H_ (DMSO-*d*_6_) = 2.50] [31] UV/Vis spectra were recorded on a Cary Varian 60 spectrometer.

All RCM experiments were performed twice unless otherwise noted: A mixture of the catalyst (1.90 µmol, 5 mol %, **2**: 1.56 mg; **3**: 1.61 mg; **4**: 1.17 mg; **5a**: 1.74 mg; **5b**: 1.48 mg; **6a**: 1.55 mg; **6b**: 1.60 mg), a supramolecular additive (1.90 µmol, 5 mol % to substrate), when applicable, and the substrate (38.0 µmol, **7**: 8.00 µL, 9.43 mg; **8**: 9.00 µL, 8.95 mg) in 830−850 µL D_2_O as the solvent was stirred at certain temperature and a constant stirring rate. An aliquot (150–300 µL) was withdrawn, diluted with MeOD-*d*_4_ to 600 µL, and directly analysed by ^1^H-NMR spectroscopy.

NMR titration experiments of the guests HPCy_3_^+^, *N*-tosyldiallylamine (**7**) and Cy_3_P=O with sulfocalixarenes **1** as hosts: The titration experiments were performed at least in duplicate using a standard “constant guest” method in 600–640 µL D_2_O with addition of 7–10 µL MeOD-*d*_4_ or 10 µL DMSO-*d*_6_ as internal standard (δ_H_ (MeOD-*d*_4_) = 3.31 ppm; δ_H_ (DMSO-*d*_6_) = 2.50 ppm) at room temperature. All H-atoms of the guests HPCy_3_^+^, *N*-tosyldiallylamine (**7**) and Cy_3_P=O were evaluated using MestRe-C [32] and MestReNova [33]. *K*_ass_ and log *K* values of the receptors were obtained by analysising the course of the chemical shifts of the protons of the guest HPCy_3_^+^, *N*-tosyldiallylamine (**7**) and Cy_3_P=O using the program HypNMR2008 [34,35].

## 4. Conclusions

In conclusion, we showed the catalytic efficiency of several commercially available *Grubbs*-type catalysts in standard ring closing metathesis reactions performed in pure water and under aerobic conditions. Supramolecular additives like sulfocalixarenes **1** can increase the catalytic activity. We investigated the type of interactions between the involved reaction components in metathesis reactions in water, *i.e.*, *Grubbs* catalyst, the substrate tosyldiallylamine **7**, and the supramolecular additives, sulfocalixarenes **1**. Sulfocalixarenes **1** do not have a micelle character, but nevertheless, supramolecular interaction with *Grubbs* precatalyst **2** lead to a kind of non-covalently formed micellar solution. In sulfocalixarene **1** assisted metathesis reactions, the conversion parallels the stirring speed clearly, indicating that the calixarenes additionally act as a mass transfer catalyst. We showed that our aqueous metathesis reaction is not a pure heterogeneous reaction, but also a fractional amount of the reactants participates in the catalytic reaction under homogeneous conditions. To provide a better insight in the intermolecular interactions, complexation binding studies in water of catalyst species, substrate **7**, and sulfocalixarene **1** were performed. The results confirm the assumption that sulfocalixarenes **1** can act as phosphine scavengers by complexation of HPCy_3_^+^, as well as Cy_3_P=O, while the *K*_ass_ value of protonated species is 10^3^-times higher. Complexation studies of the substrate as guest show different complex conformations, dependent of the cavity’s size, which we supported by MMFF calculations.

## Supplementary Materials

Supplementary Materials available: binding isotherm NMR titration experiments of HPCy_3_^+^ as guest, NMR and UV/vis spectra in methanol for the detection of catalytic species, representative NMR spectra for RCM reaction in water. Supplementary materials can be accessed at: http://www.mdpi.com/1420-3049/20/10/19130/s1.
