# Supplementary material for: Olefin Metathesis Reaction in Water and in Air Improved by Supramolecular Additives
Source: Molecules. 2015 Oct 21;20(10):19130–41. doi: 10.3390/molecules201019130 (PMC6332346; doi:10.3390/molecules201019130)
Supplement: Supplementary file 1 [file molecules-20-19130-s001.pdf]

Supplementary Materials

1. General Information

All chemicals, precatalysts **2–6**, sulfocalixarene **1** and substrate Diethyl diallylmalonate **9** were purchased from Acros®, Aldrich®, Merck® or VWR® and used without further purification, unless otherwise specified. All solvents were distilled on the rotary evaporator before appliance. Substrate *N*-Tosyldiallylamine **7** was synthesized according to literature procedure [1]. All NMR spectra were measured at room temperature (298 K) on a BRUKER Avance 400 spectrometer. Chemical shifts (δ) are expressed in ppm and either refers to not-deuterated amount of used solvents [δ_H ($CDCl_3$) = 7.26, δ_H (D_2O) = 4.79, δ_H (MeOD-d_4) = 3.31, δ_H (DMSO-d_6) = 2.50] [2]. UV/vis spectra were detected via Cary Varian 60 spectrometer. Catalysis reactions were stirred via microplate shaker IKA® MS 3 basic.

2. ^{1}H-NMR Spectra of RCM Substrates and Products

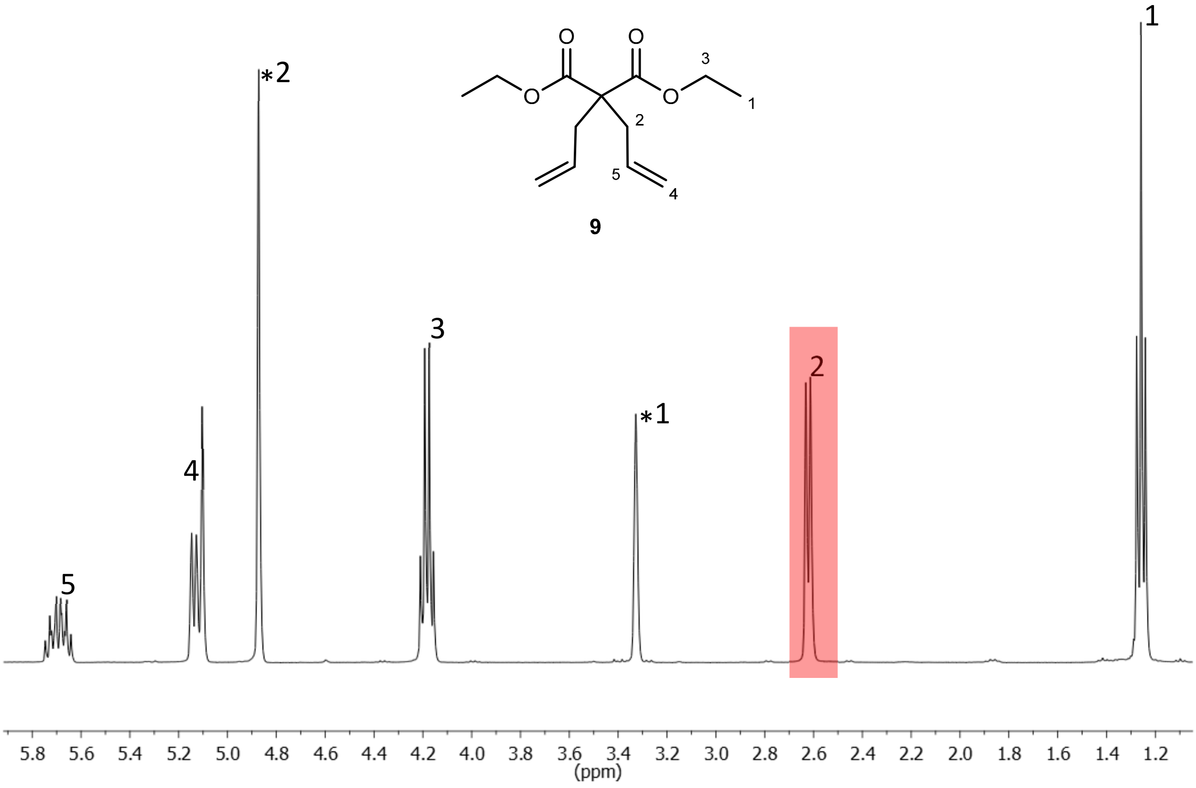

Figure S1. Extract of ^{1}H-NMR spectra (rt, 400.13 MHz, D_2O/MeOD-d_4) of RCM substrate **9**. *1 = MeOH; *2 = H_2O.

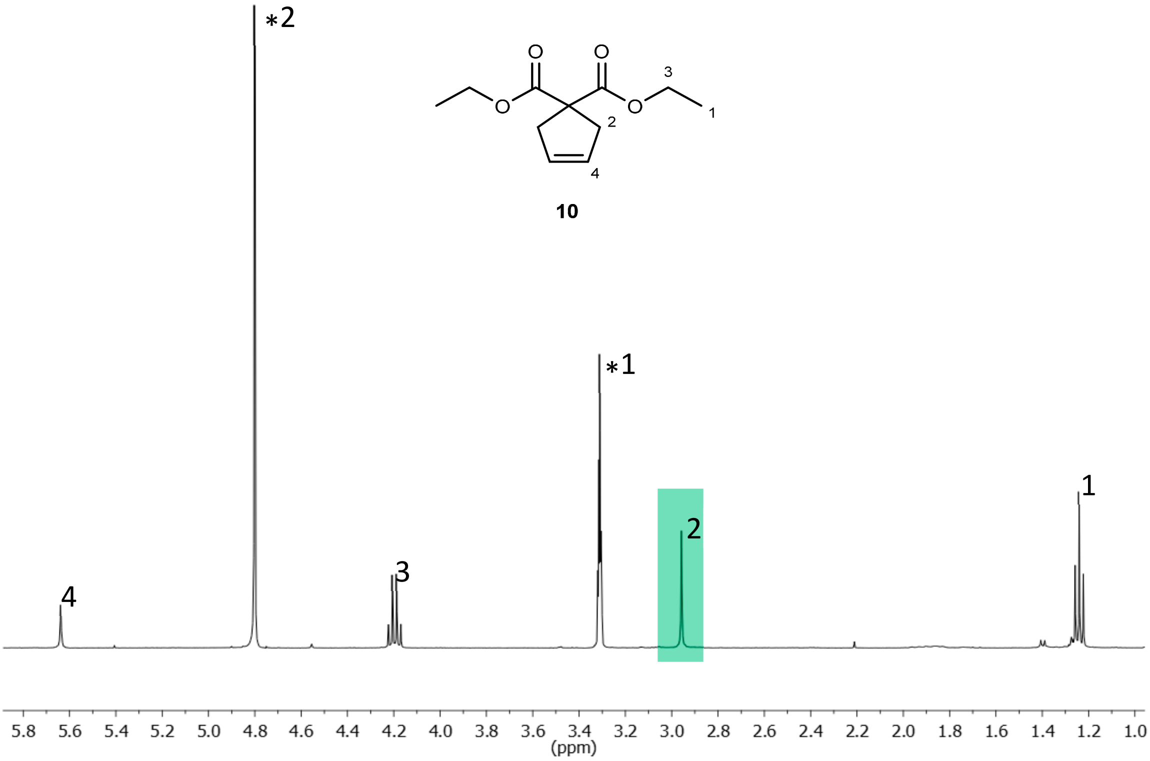

Figure S2. Extract of ^{1}H-NMR spectra (rt, 400.13 MHz, D_2O/MeOD-*d*$_4$) of complete conversion of substrate **9** to product **10** using 5 mol % precatalyst **5a**. *1 = MeOH; *2 = H_2O.

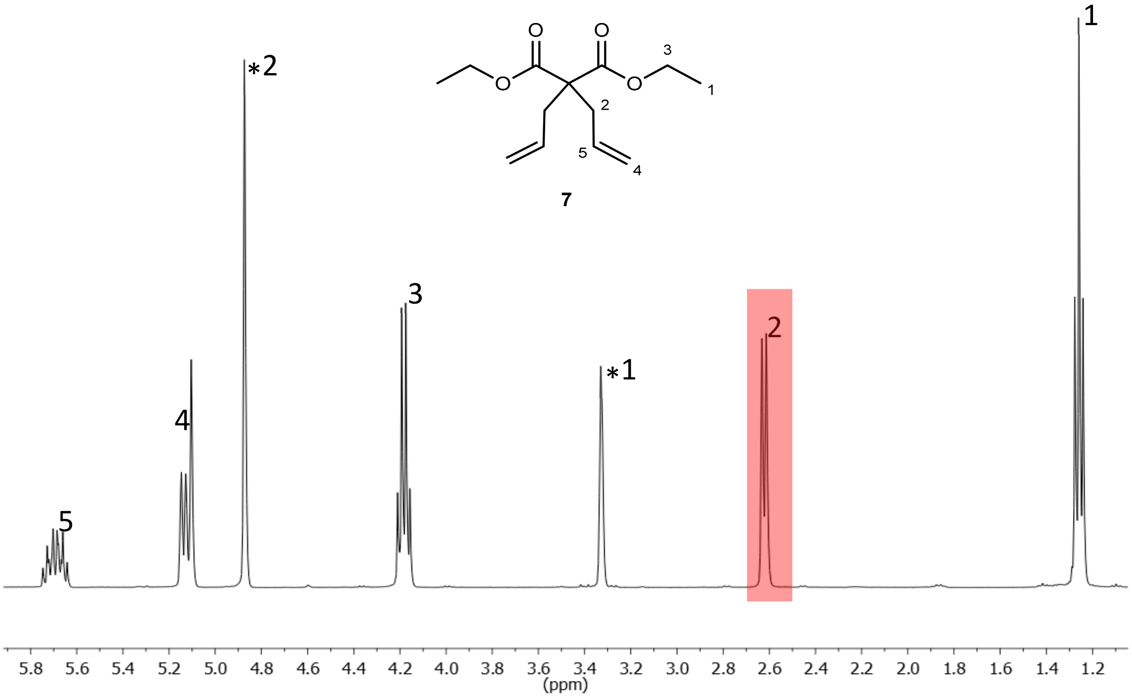

Figure S3. Extract of ^{1}H-NMR spectra (rt, 400.13 MHz, $CDCl_3$) of RCM substrate **7**. *1 = MeOH; *2 = H_2O.

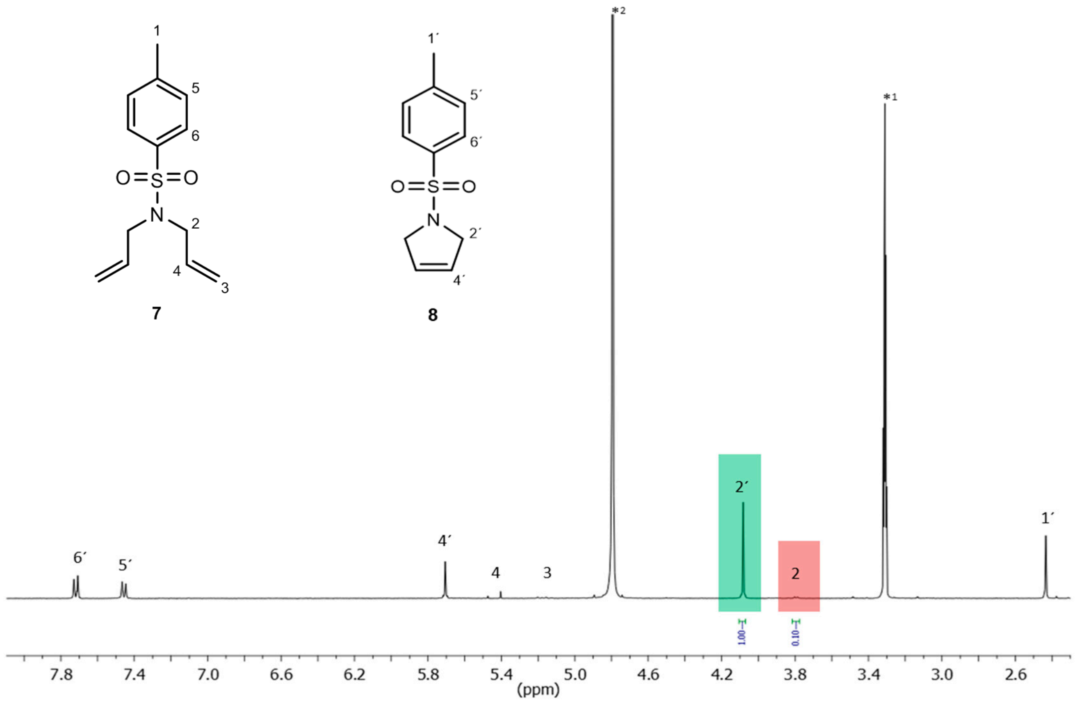

Figure S4. Extract of ^{1}H-NMR spectra (rt, 400.13 MHz, D_2O/MeOD-d_4) of RCM of substrate **7** to 91% product **8** using 5 mol % catalyst **4**. *1 = MeOH; *2 = H_2O.

3. Spectra of Solubilisation Experiments

Procedure: A mixture of the catalyst **2** (5 mg, 6.08 µmol), and a supramolecular additive **1** (7.27 mg, 6.08 µmol), when applicable in D_2O or MeOD (1 mL) as the solvent was stirred at room temperature and a constant stirring rate of 1000 rpm with exclusion of light. After stirring for a certain time, the mixture was analyzed by ^{1}H-NMR spectroscopy.

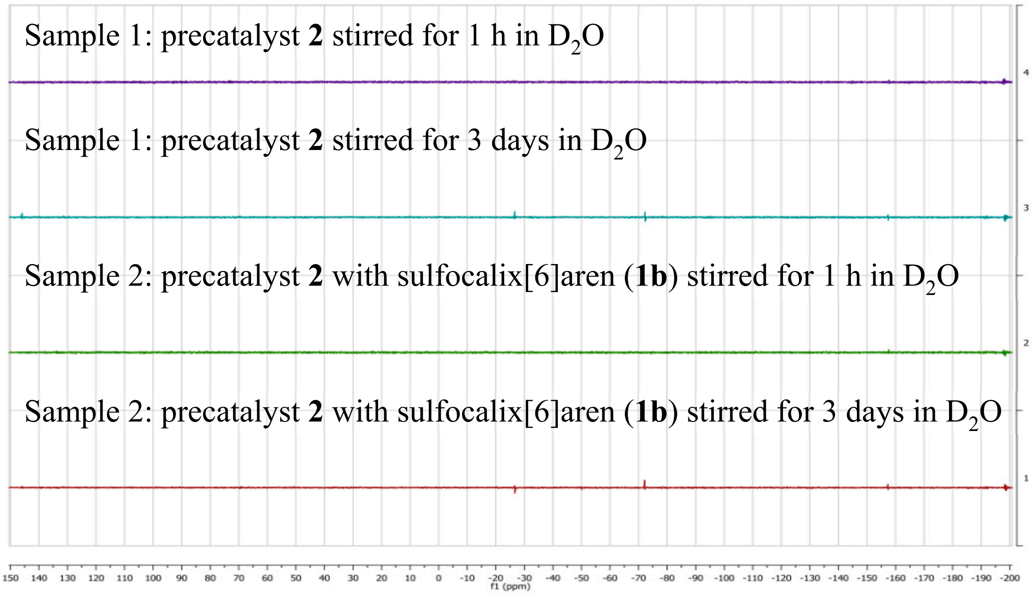

Figure S5. $^{31}P\{^1H\}$ NMR measured in D_2O.

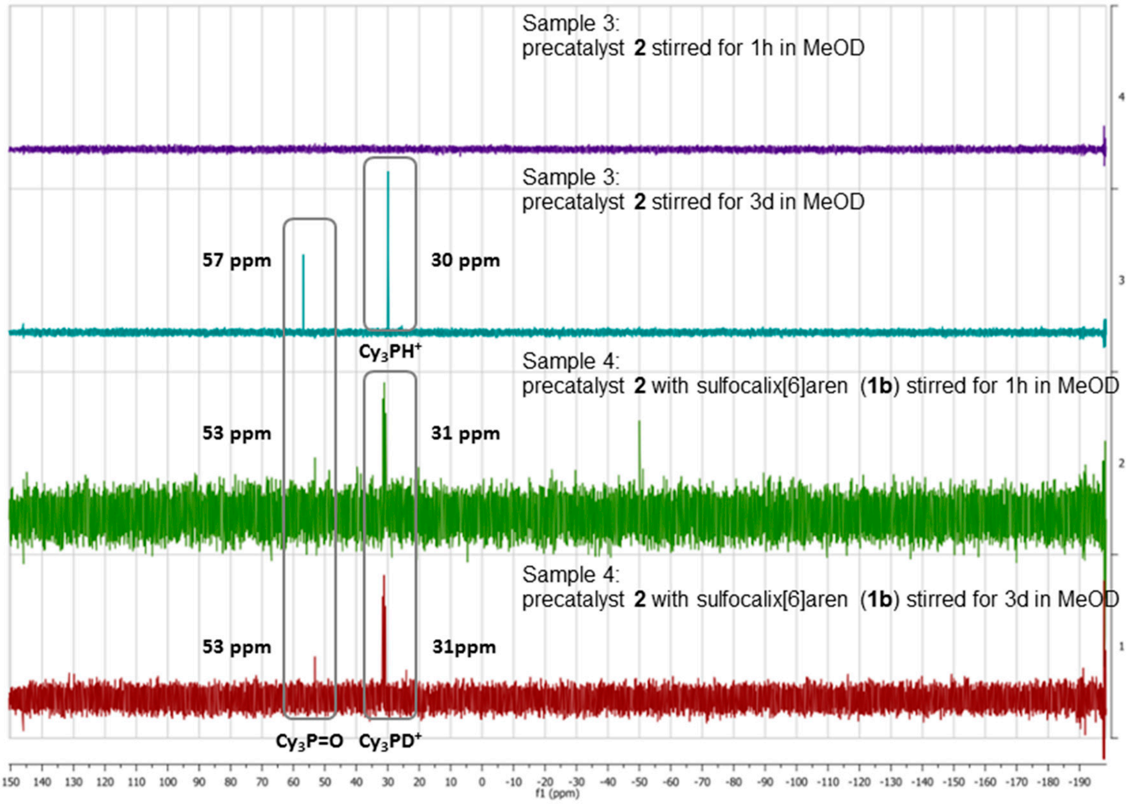

Figure S6. $^{31}P\{^1H\}$ NMR measured in MeOD.

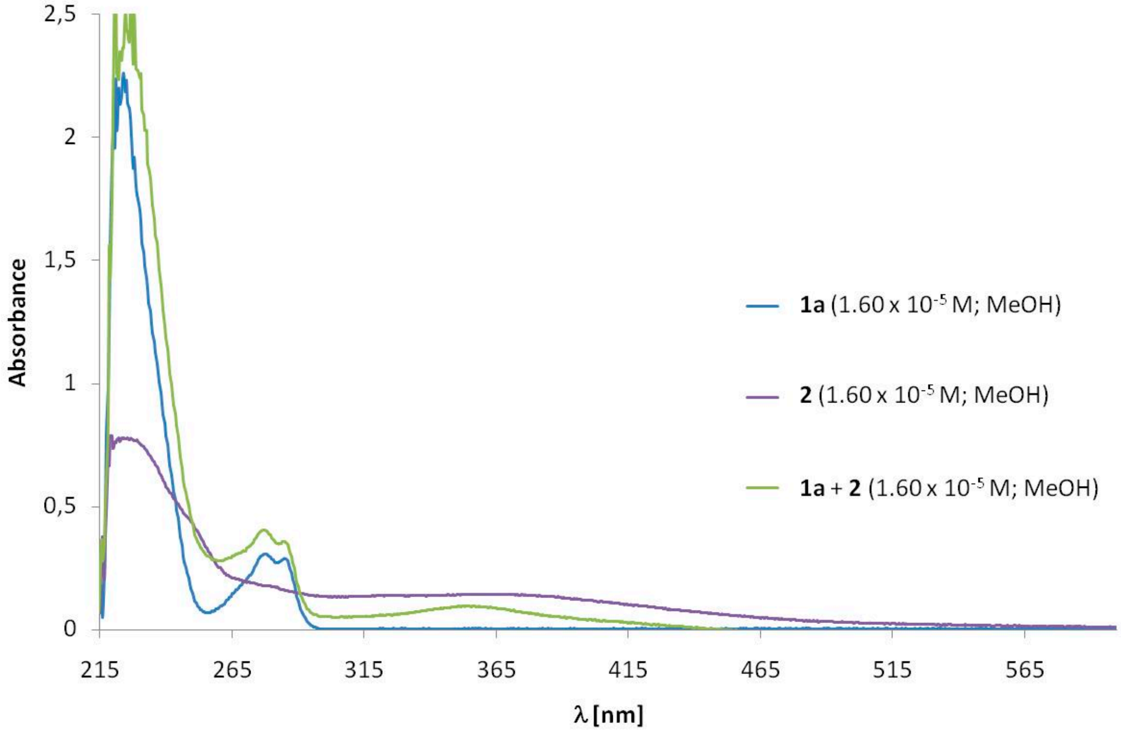

Figure S7. UV/vis spectra of precatalyst **2** and additive **1** in MeOH.

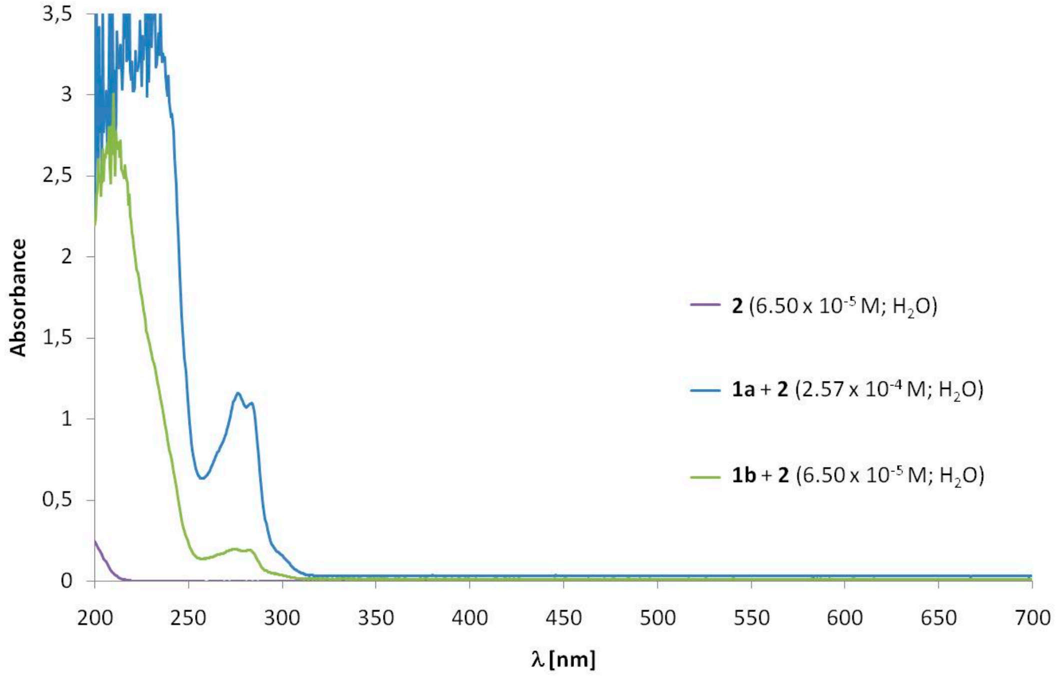

Figure S8. UV/vis spectra of precatalyst **2** and additive **1** in H_2O.

4. Binding Studies

4.1. $[Cy_3PH]^+[BF_4]^-$ with Sulfocalixarenes **1**

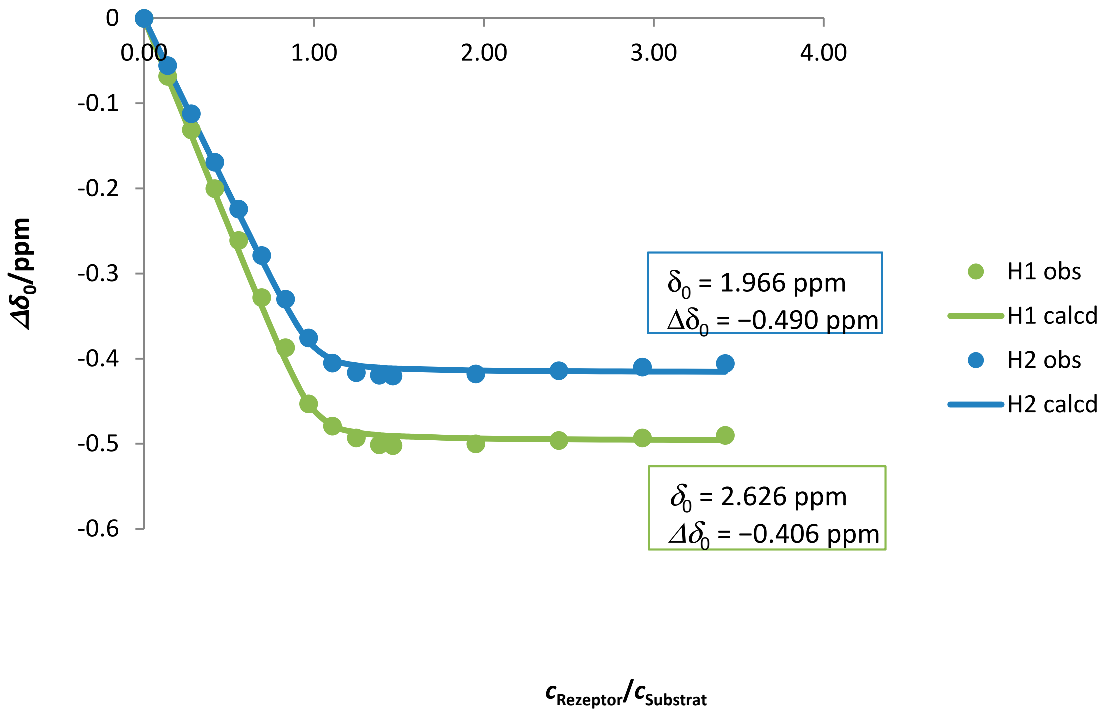

Figure S9. Binding isotherm of receptor **1a**: Plot $\Delta\delta_0$ against $c_{receptor}/c_{substrate}$ (Dots: experimental measured chemical shift; solid line: fit of the experimental data using a 1:1-binding model; $\Delta\delta = \delta_{receptor/substrate} - \delta_{substrate}$).

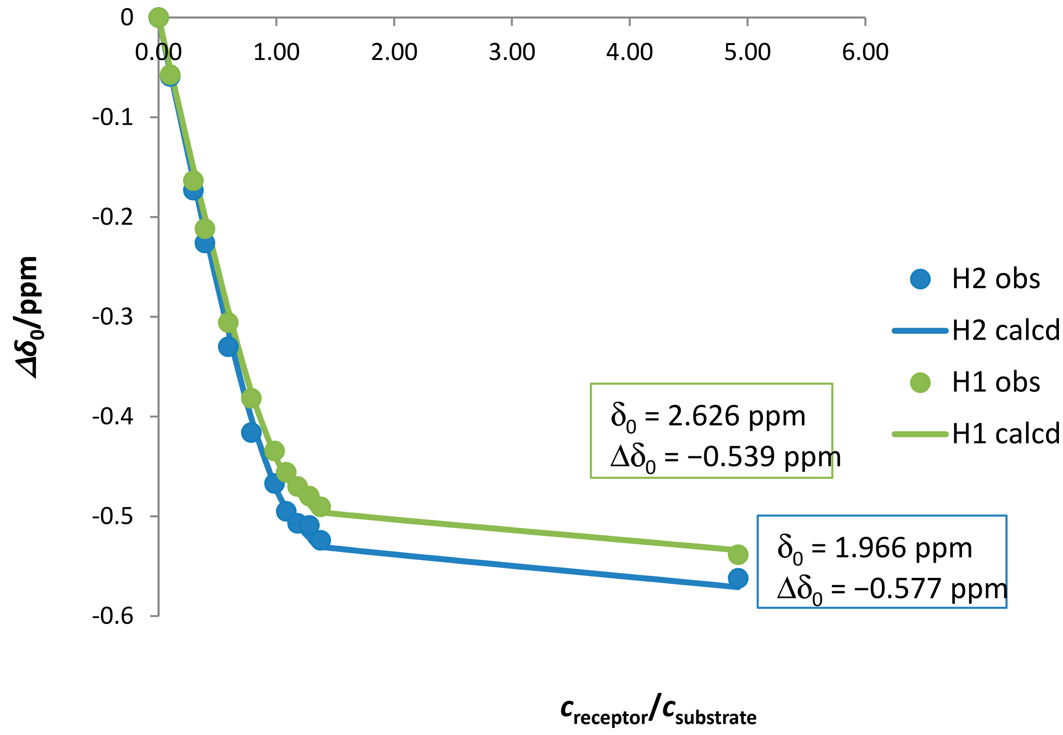

Figure S10. Binding isotherm of receptor **1b**: Plot $\Delta\delta_0$ against $c_{receptor}/c_{substrate}$ (Dots: experimental measured chemical shift; solid line: fit of the experimental data using a 1:1-binding model; $\Delta\delta = \delta_{receptor/substrate} - \delta_{substrate}$).

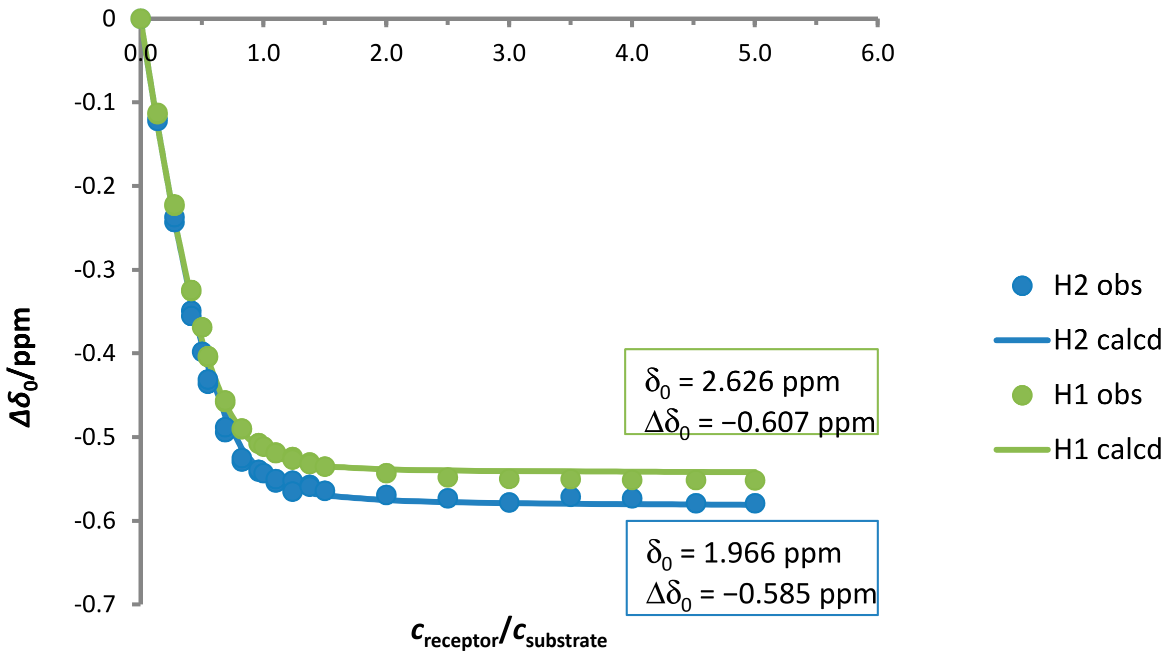

Figure S11. Binding isotherm of receptor **1c**: Plot $\Delta\delta_0$ against $c_{receptor}/c_{substrate}$ (Dots: experimental measured chemical shift; solid line: fit of the experimental data using a 1:2-binding model; $\Delta\delta = \delta_{receptor/substrate} - \delta_{substrate}$).

4.2. Cy_3P=O with Sulfocalixarenes **1b**

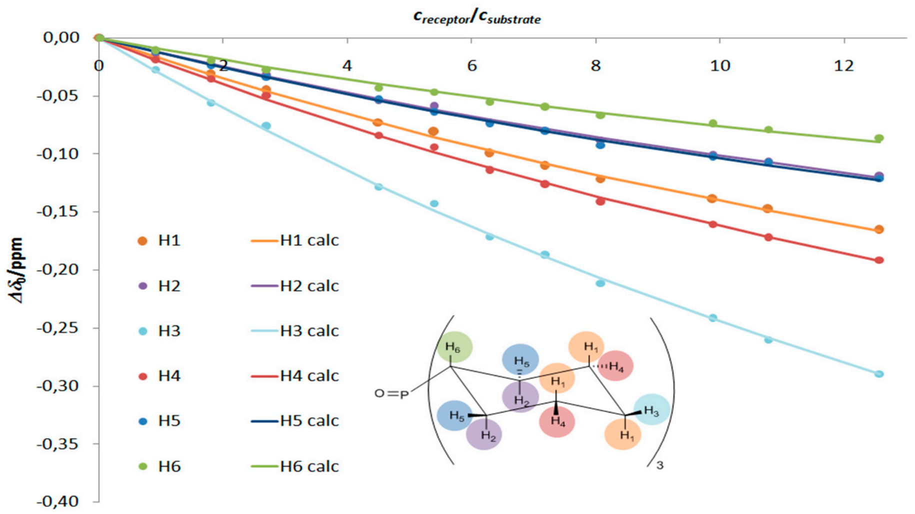

Figure S12. Binding isotherm of receptor **1b** in D_2O: Plot $\Delta\delta_0$ against $c_{receptor}/c_{substrate}$ (Dots: experimental measured chemical shift; solid line: fit of the experimental data using a 1:1-binding model; $\Delta\delta = \delta_{receptor/substrate} - \delta_{substrate}$).

Table S1. Association constant, observed chemical shifts and complexation induced chemical shift (CIS) for the binding process of receptor **1b** with Cy_3PO as guest in D_2O.

K_{Ass} = 28.8 ± 1.15 M^{-1}	H^1	H^2	H^3	H^4	H^5	H^6
δ_0 (ppm)	1.2688	1.3840	1.7218	1.8109	1.8692	2.0092
$\Delta\delta_0$ (ppm)	−0.1649	−0.1172	−0.2852	−0.1974	−0.1178	−0.0850
$\Delta\delta_{calc}$ (ppm)	−0.5458	−0.3924	−0.9371	−0.6679	−0.4035	−0.2941

$\Delta\delta_0 = \delta_{RS} - \delta_0$ determined by NMR Titration experiments measured in D_2O and MeOD as internal standard; $\Delta\delta_{calc}$ complexation induced shift, calculated by extrapolation with HypNMR.

4.3. Substrate **7** *with Sulfocalixarenes* **1**

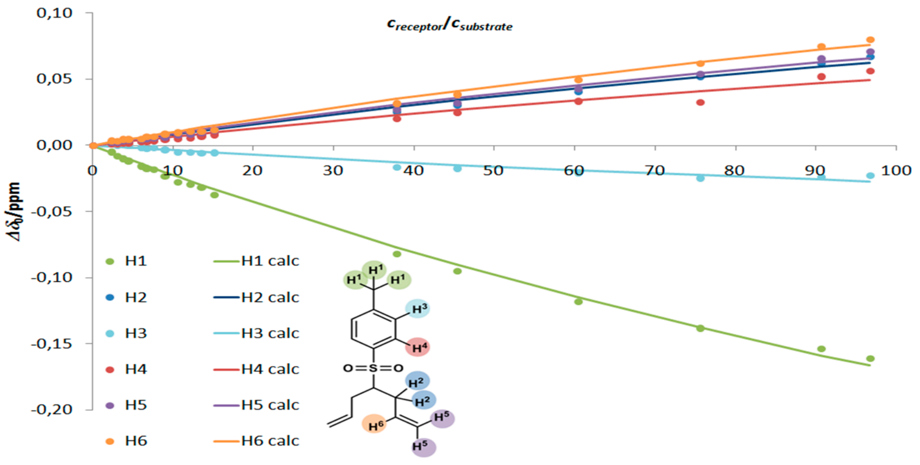

Figure S13. Binding isotherm of receptor **1a** in D_2O: Plot $\Delta\delta_0$ against $c_{receptor}/c_{substrate}$ (Dots: experimental measured chemical shift; solid line: fit of the experimental data using a 1:1-binding model; $\Delta\delta = \delta_{receptor/substrate} - \delta_{substrate}$).

Table S2. Association constant, observed chemical shifts and complexation induced chemical shift (CIS) for the binding process of receptor **1a** with **7** as guest in D_2O.

K_{Ass} = 22.4 M^{-1}	H^1	H^2	H^3	H^4	H^5	H^6
δ_0 (ppm)	2.254	3.6607	7.2923	7.5918	5.0662	5.5182
$\Delta\delta_0$ (ppm)	−0.1608	0.0673	−0.0223	0.0566	0.0713	0.0802
$\Delta\delta_{calc}$ (ppm)	−0.6564	0.2466	−0.1071	0.1949	0.2605	0.2989

$\Delta\delta_0 = \delta_{RS} - \delta_0$ determined by NMR Titration experiments measured in D_2O and DMSO as internal standard; $\Delta\delta_{calc}$ complexation induced shift, calculated by extrapolation with HypNMR.

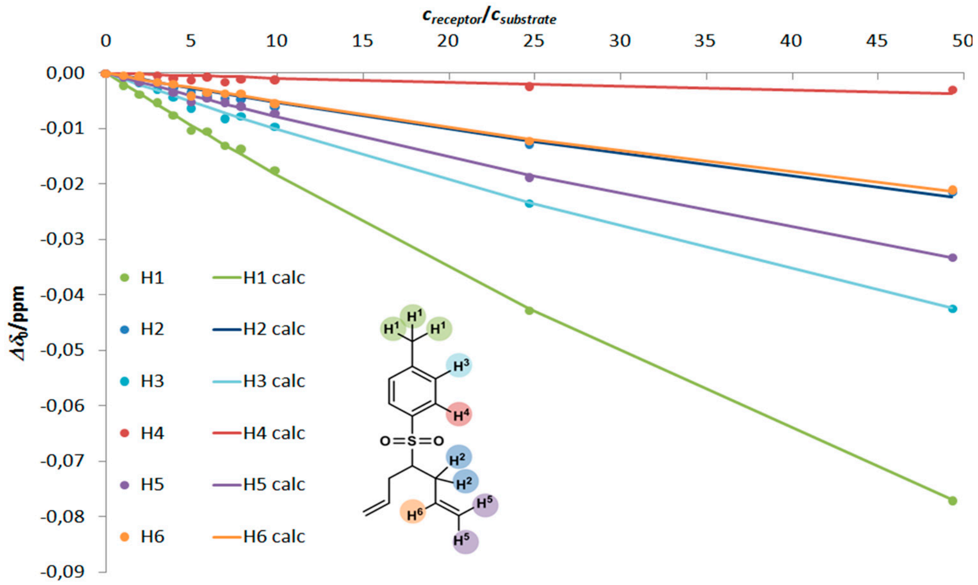

Figure S14. Binding isotherm of receptor **1b** in D_2O: Plot $\Delta\delta_0$ against $c_{receptor}/c_{substrate}$ (Dots: experimental measured chemical shift; solid line: fit of the experimental data using a 1:1-binding model; $\Delta\delta = \delta_{receptor/substrate} - \delta_{substrate}$).

Table S3. Association constant, observed chemical shifts, and complexation induced chemical shift (CIS) for the binding process of receptor **1b** with **7** as guest in D_2O.

K_{Ass} = 36.3 M^{-1}	H^1	H^2	H^3	H^4	H^5	H^6
δ_0 (ppm)	2.2536	3.6531	7.2939	7.5710	5.0664	5.5180
$\Delta\delta_0$ (ppm)	−0.0772	−0.0214	−0.0424	−0.0229	−0.0332	−0.0211
$\Delta\delta_{calc}$ (ppm)	−0.3951	−0.1146	−0.2171	−0.0189	−0.1708	−0.1101

$\Delta\delta_0 = \delta_{RS} - \delta_0$ determined by NMR Titration experiments measured in D_2O and DMSO as internal standard; $\Delta\delta_{calc}$ complexation induced shift, calculated by extrapolation with HypNMR.

References

1. So, C.M.; Kume, S.; Hayashi, T. Rhodium-Catalyzed Asymmetric Hydroarylation of 3-Pyrrolines Giving 3-Arylpyrrolidines: Protonation as a Key Step. *J. Am. Chem. Soc.* **2013**, *135*, 10990–10993.
2. Fulmer, G.R.; Miller, A.J.M.; Sherden, N.H.; Gottlieb, H.E.; Nudelman, A.; Stoltz, B.M.; Bercaw, J.E.; Goldberg, K.I. NMR chemical shifts of trace impurities: Common laboratory solvents, organics, and gases in deuterated solvents relevant to the organometallic chemist. *Organometallics* **2010**, *29*, 2176–2179.
